# Gambling Progression in Young Adults Following Online Casino-Gambling Legalization in Switzerland

**DOI:** 10.1007/s10899-025-10435-6

**Published:** 2025-09-27

**Authors:** Alexander Tomei, Marion Bieri, Clément Porchet, Olivier Simon

**Affiliations:** Centre for Excessive Gambling, Addiction Medicine, Lausanne University Hospital and University of Lausanne, Avenue de Morges 10, Lausanne, CH-1004 Switzerland

**Keywords:** Gambling, Emerging adults, Males, Post-policy impact, Switzerland

## Abstract

This study investigates gambling behaviors among young Swiss males four years after the legalization of online casino gambling in Switzerland. A total of 2,349 conscripts aged 18 to 24 years, residing in the French-speaking region of the country, completed a paper-and-pencil questionnaire assessing their participation in land-based and online gambling, as well as problem gambling. Overall, 70.6% reported having gambled at least once in their lifetime, and 49.8% had gambled in the past 12 months. Among past-year gamblers, 58.3% engaged exclusively in land-based gambling, 34.4% were mixed (land-based and online) gamblers, and 7.3% gambled exclusively online. The majority (74.7%) accessed online gambling via smartphone or tablet. Among past-year gamblers, 4.4% met the criteria for problem gambling, representing 2.1% of the total sample. Problem gambling was associated with more frequent gambling, engagement across both online and land-based platforms, participation in a greater variety of games, and involvement in online casino gambling. In conclusion, four years post-legalization, we observe increased gambling participation, a shift from land-based to online gambling, and a slight, non-significant increase in problem gambling. Continued monitoring of gambling behaviors in this population is essential to detect and respond promptly to potential increases in problem gambling.

## Introduction

Over the past two decades, gambling activities—particularly through digital platforms—have expanded significantly, paralleling developments in telecommunications. In Western countries facing industrial decline, gambling has increasingly been legitimized as a source of public revenue.

Switzerland exemplifies this trend. Since the 1923 Lottery Law, the gambling sector has steadily grown, with casino gambling legalized in 1998. Online lottery games emerged in the early 2000s, culminating in the 2019 Federal Act on Gambling (Swiss Confederation, 2017), which legalized online casino gaming for the first time. This reform allowed Swiss land-based casinos to operate online while blocking foreign platforms, under the rationale that only domestic operators can ensure adequate regulation and player protection.

A core feature of Swiss regulation is the legal requirement for gambling operators—both offline and online—to implement social responsibility measures (Carlevaro et al., 2017; Thompson, 2007), including identifying and excluding individuals exhibiting problematic gambling behaviors. Despite these provisions, the rise of online gambling has raised substantial public health concerns. Online gambling removes spatial, temporal, and monetary constraints, allowing continuous access via mobile devices. Digital transactions may obscure the perception of financial loss, while the private nature of play reduces social scrutiny. Additionally, online gambling platforms often incorporate design features that accelerate reward cycles and employ aggressive marketing strategies, both of which heighten addiction risks (García-Pérez et al., 2024; Griffiths & Auer, 2012; Hing et al., 2018).

Although current evidence does not show a direct causal link between online gambling and increased prevalence of gambling disorders in the general population (Wood et al., 2012), a stronger association has been observed among emerging adults (Arnett, 2000; Sussman & Arnett, 2014), particularly males and online poker players (Abbott et al., 2014). Online gamblers generally show higher rates of problematic behaviors compared to land-based gamblers (Gainsbury et al., 2015; Williams & Wood, 2007), with younger demographics at elevated risk (Chóliz, 2016; Hing et al., 2015; McCormack et al., 2014; Petry & Weinstock, 2007; Potenza et al., 2011; Wijesingha et al., 2017). Moreover, problem gambling is often linked to participation in a greater variety of gambling activities (Binde et al., 2017; Gooding & Williams, 2024; Holtgraves, 2009; Kessler et al., 2008; LaPlante et al., 2014; Mazar et al., 2020).

In Switzerland, a previous study found that 56.1% of males aged 18–22 had gambled in the past year, with 16% engaging in online gambling (Tomei et al., 2015). A follow-up in 2019 reported a decline in land-based gambling but an increase in online gambling from 16% to 24%. Among past-year gamblers, 17.6% were at-risk and 3.6% were classified as having a gambling problem, with online gambling more strongly associated with problem gambling (Tomei et al., 2022).

This study aims to evaluate the post-legislation impact of the 2019 Federal Act by replicating key measures from the 2019 survey. The goal is to examine how gambling behaviors—especially online—have evolved in response to regulatory and technological changes. Special attention is given to online gambling patterns (e.g., frequency, game types, devices used) and their association with problem gambling. To attain this goal, the present study was a direct replication of the 2019 survey on the same population (Tomei et al., 2022). However, the replication included two differences compared to the previous survey. First, due to recruitment delays caused by the closure of the recruitment center during the COVID-19 pandemic, this edition also included participants aged 23 and 24, whereas the previous study included participants aged 18–22 years. Second, the questionnaire section concerning online gambling behaviors incorporated the possibility of reporting bets placed on cryptocurrencies. We hypothesize increased prevalence of gambling, particularly online, and anticipate emerging associations between online problem gambling and factors such as game variety, platform type, and gambling frequency—associations not observed in 2019.

## Methods

### Sample

Paper-and-pencil questionnaires were administered to 2,618 conscripts of the Swiss Army. These conscripts were all Swiss nationals residing in the French-speaking region of Switzerland. In Switzerland, all men who have reached the age of majority are required to participate in a mandatory two-day recruitment program before their 25th birthday. The program primarily includes physical and psychological assessments, along with informational sessions. A total of 2,571 participants completed the questionnaire, yielding a response rate of 98.2%. Of these, 222 participants (8.6%) were excluded from the sample due to age-related under-representation affecting weighting, being female, residing abroad, or having missing values on at least one variable of interest. As a result, the final sample consisted of 2,349 participants, all male, aged 18–24 years (M = 20.6 years; Md = 20 years). The majority of participants were single (94.8%), 4.3% were not married but were in a cohabiting relationship, and the remaining participants (0.9%) were either married, divorced, separated, or widowed. Approximately 45% of the sample were employed, 38% were students, and 13% were unemployed (with 4% engaged in other activities). In terms of education, 16.8% of respondents had no certificate or only a compulsory school certificate, 41% had completed vocational training, 41% had a high school or tertiary level degree, and 1% reported other types of qualifications.

### Procedure

The questionnaires were administered prior to a problem gambling prevention class conducted at a Swiss Army recruitment center. The data collection took place between August 2023 and January 2024. Participants completed the questionnaires in a 150-seat auditorium, divided into groups of conscripts, with group sizes ranging from about 60 to 140 individuals. The administration of the questionnaires was carried out by a prevention team from a problem gambling prevention center, as well as by trained military personnel. Completing the questionnaire took approximately 15 min.

### Questionnaire

The cover page of the questionnaire provided information about the study’s purpose, voluntary participation, and ethical standards, including an informed consent statement. The questionnaire consisted of three sections. The first section, applicable to all participants, gathered socio-demographic data (e.g., age, sex, marital status, education) and included questions defining gambling and asking whether participants had ever gambled and whether they had gambled in the past 12 months. Responses to these filter items determined the section participants were directed to. Those who reported past-year gambling proceeded to section two, which examined gambling frequency, income, gambling debts, offline and online gambling types, and problem gambling severity using the 9-item Problem Gambling Severity Index (PGSI; Ferris & Wynne, 2001). The PGSI items were adapted into French and used a 4-point response scale ranging from 0 (Never) to 3 (Always). Scores were interpreted as follows: 0 = Non-problem gambling; 1–2 = Low-risk gambling; 3–7 = Moderate-risk gambling; 8 or more = Problem gambling.

The third section was for lifetime and past-year non-gamblers. It mainly served to occupy non-gamblers while gamblers completed section two, ensuring equal completion time for all participants. Responses from section three were not relevant to the study’s main focus.

### Weighting and Analyses

To ensure that the sample accurately reflected the age distribution of Swiss men aged 18 to 24 residing in the French-speaking region of Switzerland, data were weighted based on the Swiss population statistics (Swiss Federal Statistical Office, 2025). Descriptive analyses were conducted to explore gambling behaviors and the prevalence of problem gambling. Subsequently, cross-tabulation analyses were performed to examine the relationship between sociodemographic characteristics, gambling behaviors, and problem gambling. To ensure adequate group sizes in the contingency tables, the PGSI scores were dichotomized into two categories: Non-problem gambling (No problem and Low risk), At-risk/Problem gambling (Moderate risk and Problem gambling). All analyses related to gambling behaviors were based on past-year gambling. A significance level of *p* ≤ 0.05 was applied with Bonferroni correction for families of tests with multiple comparisons.

## Results

### Gambling Prevalence

Of the 2,349 participants, 29.4% (*n* = 721) reported that they had never engaged in gambling. In contrast, 20.9% (*n* = 451) indicated that they had gambled at least once in their lifetime but not within the 12 months prior to the survey, while 49.8% (*n* = 1,177) were identified as past-year gamblers.

### Mode of access to gambling and income

The vast majority of past-year gamblers (92.7%) had engaged in land-based gambling, while 41.7% had gambled online. Among past-year gamblers, 58.3% had only participated in land-based games, while 34.4% had gambled both land-based and online. Finally, 7.3% had engaged in online gambling exclusively. When these proportions are scaled to the total sample, they correspond to 28.9%, 17.1%, and 3.6%, respectively.

Regarding net monthly income, 18.5% of participants reported having no income in the 12 months prior to the survey. The median monthly net income was CHF 2,000, with 27.8% of respondents earning more than CHF 4,000.

###  Offline and Online Gambling

The gambling activities both off-line and online during the 12 months prior to the survey are presented in Table 1. Individuals may be represented in more than one category. The findings indicate that online gambling was more frequent than land-based gambling. Specifically, 14.3% of respondents engaged in online gambling at least once a week, compared to 9.1% for land-based gambling.

**Table 1:** Offline and online past-year gambling

*Variables*	% Offline		% Online	
Gambling frequency	(n = 1,081)		(n = 481)	
* Daily*	0.8		1.8	
* 2–6 times per week*	4.2		6.6	
* Once per week*	4.1		5.9	
* 1–3 times per month*	15.1		22.4	
* 6–11 times per year*	19.6		23.0	
* 1–5 times per year*	56.1		39.6	
Type of game	(n = 1,085)		(n = 482)	
* Scratch cards*	61.3		11.7	
* Roulette*	50.9		33.2	
* Black jack*	35.1		29.5	
* Sports betting*	34.1		51.2	
* Slot machines*	28.1		19.6	
* Poker*	22.4		14.5	
* Lottery*	18.1		12.5	
* Lottery on EGMs*	4.5		2.9	
* Scratch cards on EGMs*	4.3		-	
* Stock exchange*,* daily trading*	4.1		14.9	
* Craps*,* dice games*	3.5		1.7	
* Horse race betting*	3.1		2.5	
* Games-of-skill betting*	2.4		1.0	
* Other*	1.3		2.2	
Variety of games	(n = 1,085)		(n = 482)	
* 1*	32.2		53.7	
* 2*	23.0		20.8	
* 3*	19.3		12.6	
* 4 or more*	25.2		12.9	
Gambling locations	(n = 1,085)			
* Vending points*	62.2		-	
* Casinos*	62.8		-	
* Private locations*	25.8		-	
* Bars*,* restaurants*	11.5			
* Non-official back rooms*	4.5		-	
* Other locations*	12.1		-	
Form of payment	(n = 1,085)			
* Cash*	84.7		-	
* Credit card*	35.9		-	
* Pre-paid card*	5.6		-	
* E-banking*	4.1		-	
* Bank transfer*	3.7		-	
* Deposit*	1.6		-	
* Postal remittance slips*	0.2		-	
Electronic devices used for gambling			(n = 482)	
* Smartphone/Tablet*	-		74.7	
* Personal computer*	-		43.7	
* Computer at work*	-		2.2	
Money spent on gambling	(n = 1,002)		(n = 422)	
* None*	2.7		1.9	
1 to 50 CHF	64.5		66.5	
51 to 200 CHF	19.9		18.9	
201 to 500 CHF	8.3		9.0	
501 CHF or more	4.5		3.7	

Among land-based activities, scratch cards were the most common (61.3%), followed by roulette (50.9%), blackjack (35.1%), and sports betting (34.1%). Other popular land-based games in this sample included slot machines (28.1%), poker (22.4%), and the lottery (18.1%). In contrast, sports betting was the most prevalent form of online gambling (51.2%). Online casino games, such as roulette (33.2%), blackjack (29.5%), online slot machines (19.6%), and poker (14.5%), were also relatively common.

Furthermore, land-based gambling appeared to involve a broader variety of game types compared to online gambling. The data revealed that about 45% of land-based gamblers participated in three or more different games, whereas only slightly over 25% of online gamblers did the same.

Lottery vending locations (62.2%) and casinos (62.8%) were the most frequently visited land-based gambling venues. Private locations (25.8%) and bars and restaurants (11.5%) were also notable for their frequency of visits.

Cash was the dominant payment method for land-based gambling, used by 84.7% of participants. Credit cards were utilized by more than a third of gamblers (35.9%), while prepaid cards were less common (5.6%), with other payment methods being insignificant.

For online gambling, the majority of participants played on smartphones or tablets (74.7%), while 43.7% used personal computers. Only 2.2% reported gambling on a work computer. Regarding spending, the distribution between offline and online gambling appears to be equivalent.

#### Problem Gambling Prevalence

Problem gambling severity rates based on the PGSI are presented in Table 2. According to the PGSI, 50.5% of past-year gamblers exhibited no gambling-related problems, 28.7% were classified as low-risk gamblers, 16.5% as moderate-risk gamblers, and 4.4% were identified as persons with problem gambling. When including all participants (*N* = 2,302), both gamblers and non-gamblers, the prevalence rates were 24.6%, 14%, 8.1%, and 2.1%, respectively.

**Table 2:** Problem gambling prevalence based on the PGSI

		%
Degree of problem gambling severity		(*n* = 1,130)
*Non-problem gambling*	50.5	
*Low-risk gambling*	28.7	
*Moderate-risk gambling*	16.5	
*Problem gambling*	4.4	

## Associated Factors of Problem Gambling

### Sociodemographic Variables

Crosstab chi-square analyses revealed no association between problem gambling and past-year gamblers with occupational status, monthly net income and highest educational degree obtained. No crosstab chi-square analyses were performed with civil status because of the limited number of participants in the “married” or “widowed” categories.

#### Mode of Access, Gambling Frequency, Gambling Debts

Table 3 outlines the associations between gambling mode of access, frequency, and debts. A significant association was found between problem gambling and the mode of access to gambling (χ²(2, *N* = 1,129) = 59.572; *p* < 0.001). Specifically, the mixed online-and-land-based gambling mode was more frequently associated with at-risk/problem gambling (54.9%, 95% CI [48.1%, 61.5%]) compared to non-problem gambling (29.2%, 95% CI [26.2%, 32.4%]). Moreover, participants with at-risk/problem gambling were less likely to engage in offline gambling (at-risk/problem gambling = 36.5%; 95% CI [30.3%, 43.1%]; non-problem gambling = 63.6%; 95% CI [60.3%, 66.9%]).

**Table 3:** Association of mode of access, gambling frequency and gambling debts of past-year gamblers with problem gambling

Variables	Non-problem gambling	At-risk/Problem gambling	
	%	%	
Mode of access	(*n* = 896)	(*n* = 228)	
* Offline only*	63.6	36.5	***
* Offline and online*	29.2	54.9	
* Online only*	7.2	8.6	
Gambling frequency	(*n* = 896)	(*n* = 230)	
* Once per week or more*	5.2	24.2	***
* One to three times per month*	12.9	25.2	
* One to eleven times per year*	81.9	50.5	
Gambling debts	(*n* = 898)	(*n* = 231)	
* No*	99.2	95.2	***
* Yes*	0.8	4.8	

Note : *** *p*< 0.001

A significant association was also observed between problem gambling and gambling frequency (χ²(2, *N* = 1,129) = 116.338; *p* < 0.001). Respondents in the at-risk/problem gambling group (24.2%, 95% CI [18.9%, 30.5%]) were nearly five times more likely than those in the non-problem gambling group (5.2%, 95% CI [3.8%, 6.9%]) to engage in gambling at least once a week.

Furthermore, a significant association was identified between gambling debts and the presence of at-risk/problem gambling (χ²(1, *N* = 1,129) = 18.397; *p* < 0.001). The results indicated that the proportion of participants exhibiting at-risk/problem gambling behavior (4.8%, 95% CI [2.6%, 8.6%]) was higher among those with gambling debts compared to those without gambling issues (0.8%, 95% CI [0.4%, 1.6%]).

#### Gambling Behaviors

Table 4 outlines the associations between land-based gambling behaviors and problem gambling. We applied the Bonferroni correction for families of tests that included multiple comparisons (i.e. Type of land-based game played, Gambling locations, and Forms of payment). After the correction, adjusted significance threshold were α = 0.004 (0.05/14) for Type of land-based game played, α = 0.01 (0.05/5) for Gambling locations, and α = 0.007 (0.05/7) for Forms of payment. At-risk/Problem gambling was significantly associated with all types of land-based games except for Scratch cards, Lottery, Stock exchange and daily trading, and other sorts of games (i.e. darts, wheel of fortune, specific card games).

**Table 4:** Association of land-based gambling behaviors with problem gambling

Variables	Non-problem gambling		At-risk/Problem gambling		
	%	%			
Type of land-based game played	(*n* = 830)	(*n* = 209)			
*Scratch cards*	61.5	60.5		*ns*	
*Roulette*	44.5	72.1		***	
*Black jack*	29.8	52.6		***	
*Sports betting*	26.8	60.5		***	
*Slot machines*	24.6	39.1		***	
*Poker*	19.5	31.7		***	
*Lottery*	16.5	22.8		*ns*	
*Lottery on EGMs*	3.1	8.8		***	
*Scratch cards on EGMs*	2.3	10.9		***	
*Stock exchange*,* daily trading*	3.0	7.5		*ns*	
*Craps*,* dice games*	2.3	8.5		***	
*Horse race betting*	1.9	7.2		***	
*Games-of-skill betting*	1.5	6.4		***	
*Other*	1.1	2.3		*ns*	
Number of different land-based games played	(*n* = 830)	(*n* = 209)			
*1*	36.7	17.9		***	
*2*	25.1	17.1			
*3*	19.6	17.4			
*4 or more*	18.5	47.7			
Gambling locations	(*n* = 830)	(*n* = 209)			
*Lottery vending points*	59.8	70.5		**	
*Casinos*	58.5	77.4		***	
*Private locations*	25.8	25.1		*ns*	
*Bars and restaurants*	10.4	14.4		*ns*	
*Back rooms/Unofficial places*	3.3	8.9		**	
Forms of payment	(*n* = 830)	(*n* = 209)			
*Cash*	84.5	86.7		*ns*	
*Credit card*	34.6	41.5		*ns*	
*Pre-paid card*	5.7	6.0		*ns*	
*E-banking*	3.6	6.4		*ns*	
*Bank transfer*	2.4	9.2		***	
*Deposit*	1.1	3.8		*ns*	
*Postal remittance slip*	0.0	0.8		*ns*	
Money spent on land-based gambling	(*n* = 824)	(*n* = 206)			
*None*	9.8	13.0		***	
*1 to 50 CHF*	75.5	39.3			
*51 to 200 CHF*	12.3	29.4			
201 *CHF or more*	2.4	18.3			

Note : *ns* non-significant; *** *p* < 0.001; ** *p* < 0.01; For variables *Type of land-based game played*, *Gambling locations*, and *Forms of payment* (multiple-response items), comparisons were conducted separately for each category. For variables *Number of different land-based games played* and *Money spent on land-based gambling*, a single analysis examined their overall association

Furthermore, problem gambling was associated with several land-based gambling behaviors. Specifically, it was linked to the variety of games played (χ²(2.99, *N* = 1,039) = 82.423; *p* < 0.001). Participants with at-risk/problem gambling (47.7%, 95% CI [40.7%, 54.8%]) were significantly more likely to gamble on four or more types of games than their non-problem gambling counterparts (18.5%, 95% CI [15.9%, 21.4%]).

Additionally, problem gambling was associated with specific land-based gambling locations, including casinos (χ²(1, *N* = 1,039) = 25.793; *p* < 0.001), and backrooms/unofficial locations (χ²(1, *N* = 1,039) = 12.240; *p* < 0.01). In these locations, participants with at-risk/problem gambling were more prevalent than their non-problem gambling counterparts. No significant differences were found for other gambling venues.

Only one payment method was significantly associated with problem gambling: bank transfers (χ²(1, *N* = 1,039) = 21.792; *p* < 0.001). Its use was significantly higher among respondents with at-risk/problem gambling.

Finally, crosstab chi-square analyses revealed a significant association between the amount of money spent on gambling and the presence of problem gambling behaviors (χ²(2.99, *N* = 1,030) = 139.636; *p* < 0.001). As expected, participants identified as having at-risk/problem gambling (18.3%, 95% CI [13.4%, 24.5%]) were more likely to report spending higher amounts of money on gambling compared to their non-problem gambling counterparts (2.4%, 95% CI [1.5%, 3.8%]).

Table 5 summarizes the associations between online gambling behaviors and problem gambling status. We applied the Bonferroni correction to the tests within the Type of game played online. Thus, the significance threshold was adjusted α = 0.004 (0.05/13). Crosstab chi-square analyses on the type of online games played in the past year, showed that it was specifically the casino online games that were significantly associated with the presence of at-risk/problem gambling, namely the roulette, blackjack, poker, and online slot machines (all χ² greater than 16.0, all p-values smaller than 0.001).

**Table 5:** Association of online gambling behaviors with problem gambling

Variables	Non-problem gambling		At-risk/Problem gambling		
	%	%			
Type of online game played	(*n* = 323)	(*n* = 142)			
* Scratch cards*	11.4	11.4		*ns*	
* Sports betting*	48.5	58.4		*ns*	
* Roulette*	26.3	48.2		***	
* Black jack*	22.8	41.7		***	
* Poker*	9.8	23.9		***	
* Slot machines*	17.0	24.2		*ns*	
* Lottery*	13.3	9.4		*ns*	
* Lottery on EGMs*	3.0	2.3		*ns*	
* Stock exchange*,* daily trading*	14.5	16.5		*ns*	
* Craps*,* dice games*	1.1	2.5		*ns*	
* Horse race betting*	1.4	4.4		*ns*	
* Games-of-skill betting*	1.0	1.2		*ns*	
* Other*	2.0	2.0		*ns*	
Number of different online games played	(*n* = 323)	(*n* = 142)			
* 1*	59.9	43.1		***	
* 2*	21.3	19.0			
* 3 or more*	18.8	37.9			
Electronic devices used for gambling	(*n* = 261)	(*n* = 97)			
* Smartphone/Tablet only*	47.3	26.5		***	
* Personal computer only*	29.3	26.5			
* Both*	23.4	44.5			
Money spent on online gambling	(*n* = 322)	(*n* = 139)			
* None*	14.9	8.9		***	
* 1 to 50 CHF*	70.5	41.8			
* 51 or more*	14.6	49.3			

Note : *ns* non-significant; *** *p* < 0.001; For variables *Type of online game played* (multiple-response items), comparisons were conducted separately for each category. For variables *Number of different online games played*, *Electronic devices used for gambling*, and *Money spent on online gambling* a single analysis examined their overall association

At-risk/problem gambling was significantly associated with the number of different online games played (χ²(2, *N* = 461) = 20.123; *p* < 0.001), with individuals in the at-risk/problem gambling group engaging in a wider variety of games (37.9%, 95% CI [30.0%, 46.5%]) compared to the non-problem gambling group (18.8%, 95% CI [14.7%, 23.8%]).

Regarding the type of electronic devices used for online gambling, individuals with at-risk/problem gambling differed from their non-problem gambling counterparts (χ²(2, *N* = 358) = 17.004; *p* < 0.001): the former reported higher usage of both smartphones/tablets and personal computers for gambling (at-risk/problem gambling = 44.5%; 95% CI [34.5%, 54.8%]; non-problem gambling = 23.4%; 95% CI [18.3%, 29.3%]), whereas the latter primarily gambled via smartphones/tablets only (non-problem gambling = 47.3%; 95% CI [41.0%, 53.6%]; at-risk/problem gambling = 26.5%; 95% CI [20.7%, 39.2%]).

Finally, participants in the at-risk/problem gambling group (49.3%, 95% CI [40.7%, 57.9%]) reported significantly higher online gambling expenditures compared to their non-problem gambling counterparts (14.6%, 95% CI [10.9%, 19.1%]; χ²(2, *N* = 461) = 62.837; *p* < 0.001).

## Discussion

This study reports that approximately 71% of emerging adult males in the French-speaking region of Switzerland have gambled at least once in their lifetime, with nearly 50% gambling in the past year. This aligns with global data from Tran et al. (2024), which found a 49.1% past-year gambling rate for males across 68 countries. These findings indicate a resurgence in gambling after a period of decline, as past-year gambling rates dropped from 56.1% in 2012 (Tomei et al., 2015) to 46.7% in 2019 (Tomei et al., 2022), but increased again to 50% in 2023.

In terms of gambling formats, most participants still engaged primarily in land-based gambling, but the proportion gambling exclusively online more than tripled since 2019 (Tomei et al., 2022). This shift is likely linked to the pandemic, which limited access to physical venues, and the launch of six new online gambling platforms in Switzerland between 2020 and 2021 (Swiss Federal Gambling Commission, 2024).

Online gambling was more frequent than land-based gambling, with sports betting being the most popular online activity. In contrast, land-based gamblers preferred scratch cards, while casino games such as roulette and blackjack were equally popular across both formats. Land-based gambling was also linked to a broader variety of games, with 25% of past-year land-based gamblers engaging in four or more types of games, compared to 13% of those who gambled only online. Cash remained the primary payment method, though electronic payment methods (e.g., credit cards, bank transfers) accounted for about half of all transactions. Expenditures were similar across both gambling formats. Smartphones and tablets were the primary devices for online gambling, followed by personal computers.

Using the Problem Gambling Severity Index (PGSI), the study found that 2.1% of young men met the criteria for problem gambling, with this rate rising to 4.4% among past-year gamblers. When including at-risk gamblers, approximately 20% of past-year gamblers reported at-risk/problematic gambling, consistent with global estimates (Tran et al., 2024). Educational attainment was a significant factor, as those without a diploma or only a compulsory school education were overrepresented among persons with at-risk/problem gambling. Participants with at-risk/problem gambling also tended to gamble more frequently, incur gambling-related debt, and engage in both online and land-based gambling, consistent with previous findings (Gainsbury, 2015; Gonzalez-Roz et al., 2017). Participants with at-risk/problem gambling often gambled at lottery outlets and casinos, using electronic payment methods, particularly bank transfers for higher expenditures. Notably, they also preferred unofficial, unsupervised venues such as back rooms and unofficial locations. Unlike sports betting, lotteries and stock exchange, online casino games were strongly linked to problem gambling, thus lending support to the observation that this form of gambling is particularly harmful (Marionneau et al., 2024). At-risk/problematic gambling was also associated with using a variety of devices and higher spending. Thus, our findings together with those they align with, call for closer monitoring of online gambling - particularly online casino gambling - among young people in the coming years. For their part, regulators should ensure that early online detection of problem gambling is standardized based on stringent criteria, in order to keep the prevalence of problem gambling as low as possible.

The study examined the gambling behaviors of young males aged 18–24, four years after the 2019 legalization of online casinos in Switzerland. Its findings indicate an increase in gambling participation, especially online. However, the prevalence of problem gambling has only risen slightly compared to 2019, with no notable change in moderate-risk gambling or non-problem gambling. This suggests that the legalization of online casinos did not have a major impact problem gambling rates, likely due to the availability of unlicensed foreign gambling sites before 2019. Historical data supports this, showing no significant increase in problem gambling after the 2000 Swiss Gambling and Casino Act (Bondolfi et al., 2008). Therefore, a rise in online gambling activity does not necessarily correspond to an increase in the prevalence of problem gambling (Lind et al., 2022). However, our findings contradict those found previously elsewhere that have shown an increase of the number of gamblers entering treatment in the years following the legalization of online gambling (Chóliz, 2016). Our findings are also in contrast with data from Swiss online gambling studies conducted before (Al Kurdi et al., 2020) and during COVID-19 restrictions (Notari et al., 2023) show increased problem gambling in the general population, particularly among 18–29-year-olds.

The integration of automated, mechanical early-detection algorithms by Swiss online casinos resulted in a tripling of exclusions between 2019 and 2023 (Swiss Federal Gambling Commission, 2025), with young individuals being predominantly affected. Further, gambling behaviors such as frequency, game variety, gambling location, and spending were all significantly linked to at-risk/problem gambling. These findings are consistent with previous research (Gainsbury, 2015; Gonzalez-Roz et al., 2017; Williams et al., 2012; Zendle, 2020).

Limitations of the study include its male-only sample, and specifically young males. Further, the study lack of measurement for emerging gambling formats like hybrid sports betting and cryptocurrency gambling, which have been linked to problem gambling (Wardle & Zendle, 2021). Another limitation of the study is its reliance on self-reported questionnaires, which are subject to individual interpretation, recall bias, and social desirability bias — particularly given the group setting in which the questionnaires were administered in the present study. These factors may affect the accuracy and reliability of the data. Future research should address these areas.

In conclusion, as we expected, gambling participation among young males in Switzerland has increased since the 2019 Swiss Gambling Act, with a shift from land-based to online gambling. Online casino games were strongly linked to problem gambling. To prevent a further rise in gambling-related harm and to better manage existing risks, effective monitoring and early detection systems should be maintained or even reinforced. Prevention efforts should integrate digital tools alongside traditional public health strategies to ensure a comprehensive and timely response.

## Data Availability

The data that support the findings of this study are available upon reasonable request to the corresponding author.
